# Supplementary material for: Curcumin Improves Neurogenesis in Alzheimer’s Disease Mice via the Upregulation of Wnt/β-Catenin and BDNF
Source: Int J Mol Sci. 2024 May 8;25(10):5123. doi: 10.3390/ijms25105123 (PMC11120842; doi:10.3390/ijms25105123)
Supplement: Supplementary file 1 [file ijms-25-05123-s001.zip › R1-Supplemental tables/R1-Table S1.pdf]

Supplementary Table S1: Information of antibodies used in western blot.

Antibody	Company	Titer
anti-phospho-β-catenin	Cell Signaling Technology, USA	1:1000
anti-β-catenin	Cell Signaling Technology, USA	1:1000
anti-phospho-GSK3β	Cell Signaling Technology, USA	1:1000
anti-GSK3β	Cell Signaling Technology, USA	1:1000
anti-phospho-CREB	Cell Signaling Technology, USA	1:1000
anti-CREB	Abcam, USA	1:1000
anti-phospho-Akt	Abcam, USA	1:1000
anti-Akt	Abcam, USA	1:1000
anti-GAPDH	Cell Signaling Technology, USA	1:2000
anti-β-actin	Cell Signaling Technology, USA	1:2000
