# Supplementary material for: Curcumin Improves Neurogenesis in Alzheimer’s Disease Mice via the Upregulation of Wnt/β-Catenin and BDNF
Source: Int J Mol Sci. 2024 May 8;25(10):5123. doi: 10.3390/ijms25105123 (PMC11120842; doi:10.3390/ijms25105123)
Supplement: Supplementary file 1 [file ijms-25-05123-s001.zip › R1-Supplemental tables/R1-Table S2.pdf]

Supplementary Table S2: Information of antibodies used in immunostaining.

**Antibody**	**Company**	**Titer**
Rat monoclonal anti-BrdU antibody	Abcam, UK	1:200
Alexa Fluor 594 AffiniPure Donkey Anti-Rat lgG (H+L)	Yeasen, China	1:100
CY3-labeled anti-rat IgG antibody	Millipore, USA	1:200
Rabbit monoclonal anti-Neun antibody	Cell Signaling Technology, USA	1:200
rabbit polyclonal anti-DCX Antibody	Cell Signaling Technology, USA	1:100
Alexa Fluor 488 AffiniPure Donkey Anti-Rabbit lgG (H+L)	Yeasen, China	1:100
